# Gaboxadol Normalizes Behavioral Abnormalities in a Mouse Model of Fragile X Syndrome

**DOI:** 10.3389/fnbeh.2019.00141

**Published:** 2019-06-25

**Authors:** Patricia Cogram, Robert M. J. Deacon, Jennifer L. Warner-Schmidt, Melanie J. von Schimmelmann, Brett S. Abrahams, Matthew J. During

**Affiliations:** ^1^FRAXA-DVI, FRAXA Research Foundation, Boston, MA, United States; ^2^Centre for Systems Biotechnology, Biomedicine Division, Fraunhofer-Gesellschaft, Santiago, Chile; ^3^GEN.DDI Limited, London, United Kingdom; ^4^Institute of Ecology and Biodiversity (IEB), University of Chile, Santiago, Chile; ^5^NeuroJenic Consulting, LLC, Garden City, NY, United States; ^6^Ovid Therapeutics, New York, NY, United States; ^7^Department of Genetics and Neuroscience, Albert Einstein College of Medicine, Bronx, NY, United States; ^8^Department of Neurological Surgery and Molecular Virology, Immunology and Medical Genetics, Ohio State University College of Medicine, Columbus, OH, United States

**Keywords:** FMRP, GABA, THIP, OV101, hyperactivity, anxiety, aggression, stereotypy

## Abstract

Fragile X syndrome (FXS) is the most common inherited form of intellectual disability and autism. FXS is also accompanied by attention problems, hyperactivity, anxiety, aggression, poor sleep, repetitive behaviors, and self-injury. Recent work supports the role of γ-aminobutyric-acid (GABA), the primary inhibitory neurotransmitter in the brain, in mediating symptoms of FXS. Deficits in GABA machinery have been observed in a mouse model of FXS, including a loss of tonic inhibition in the amygdala, which is mediated by extrasynaptic GABA_A_ receptors. Humans with FXS also show reduced GABA_A_ receptor availability. Here, we sought to evaluate the potential of gaboxadol (also called OV101 and THIP), a selective and potent agonist for delta-subunit-containing extrasynaptic GABA_A_ receptors (dSEGA), as a therapeutic agent for FXS by assessing its ability to normalize aberrant behaviors in a relatively uncharacterized mouse model of FXS (*Fmr1* KO2 mice). Four behavioral domains (hyperactivity, anxiety, aggression, and repetitive behaviors) were probed using a battery of behavioral assays. The results showed that *Fmr1* KO2 mice were hyperactive, had abnormal anxiety-like behavior, were more irritable and aggressive, and had an increased frequency of repetitive behaviors compared to wild-type (WT) littermates, which are all behavioral deficits reminiscent of individuals with FXS. Treatment with gaboxadol normalized all of the aberrant behaviors observed in *Fmr1* KO2 mice back to WT levels, providing evidence of its potential benefit for treating FXS. We show that the potentiation of extrasynaptic GABA receptors alone, by gaboxadol, is sufficient to normalize numerous behavioral deficits in the FXS model using endpoints that are directly translatable to the clinical presentation of FXS. Taken together, these data support the future evaluation of gaboxadol in individuals with FXS, particularly with regard to symptoms of hyperactivity, anxiety, irritability, aggression, and repetitive behaviors.

## Introduction

Fragile X syndrome (FXS) is the most common inherited form of intellectual disability and autism with an estimated frequency of 1:4,000–5,000, affecting all ethnic groups worldwide (Gross et al., 2015). Individuals with FXS are at increased risk for a range of associated behavioral issues, including: attention problems, hyperactivity, anxiety, and many features associate with autism including motor stereotypies, social avoidance, self-injurious behavior, and aggression (Hagerman et al., 2010). Additionally, individuals with FXS are prone to comorbid medical issues including seizures, sleep disturbances, gastrointestinal difficulties, and connective tissue problems. Behavioral interventions and pharmacological management of discrete symptoms are offered to individuals with FXS, but there are currently no FDA-approved therapies to treat the syndrome as a whole.

FXS is a result of mutations in the *FMR1* gene that block the expression of the fragile X mental retardation protein (FMRP). FMRP is a ubiquitously expressed mRNA binding protein required for transport and translation of 4%–8% of synaptic proteins, and thus regulates a variety of synaptic functions (Bassell and Warren, 2008). The role of FMRP in FXS and evaluation of candidate therapeutics has been studied in large part through the use of an *Fmr1* knockout (KO) mouse model first characterized by the Dutch-Belgian Fragile X Consortium (Bakker et al., 1994). The *Fmr1* KO was generated by a targeted insertion of a neomycin cassette into exon 5 of the *FMR1* gene, resulting in a mouse that had undetectable levels FMRP protein and low levels of residual *Fmr1* mRNA (Bakker et al., 1994). The Fmr1 KO2 mouse targets a new null allele at Fmr1 generated by deletion of the promoter and first exon of Fmr1 (Mientjes et al., 2006). It is both protein and mRNA null. This model has been widely used for drug testing (Deacon et al., 2015; Cheng et al., 2017; Gaudissard et al., 2017; Dahlhaus, 2018; Leboucher et al., 2019; Tranfaglia et al., 2019), and the battery of behavioral testing has been largely focused on hyperactivity, increased sensitivity to auditory stimuli, stereotypy and deficits in learning and memory (Olmos-Serrano et al., 2011). At the cellular and circuit levels, studies of the *FXS* mice have focused largely on overactive metabotropic glutamate receptor 5 (mGluR5) signaling (Dölen et al., 2007), increased protein synthesis (Osterweil et al., 2010), and enhanced long-term potentiation (LTP; Auerbach and Bear, 2010).

A γ-aminobutyric acid (GABA)ergic hypothesis of FXS has also emerged based on observations from individuals with FXS and mice lacking an active copy of the *Fmr1* gene. GABA is the primary inhibitory neurotransmitter in the brain and signals post-synaptically through either fast ionotropic (GABA_A_) receptors or slow metabotropic (GABA_B_) receptors. Within the GABA_A_ receptor population, a further distinction is drawn between synaptic and extrasynaptic receptors that mediate phasic and tonic inhibition, respectively (Olmos-Serrano et al., 2010; Meera et al., 2011). In support of the GABAergic hypothesis, individuals with FXS show reduced GABA_A_ receptor availability (D’Hulst et al., 2015). This result from individuals with FXS is paralleled nicely by findings in a mouse model of disease.

Molecular studies have shown decreases in GABAergic machinery, including alterations in GABA receptor subunits, the GABA transporter (Rotschafer et al., 2015), and glutamic acid decarboxylase (GAD; D’Hulst and Kooy, 2007; Olmos-Serrano et al., 2010). More specifically, mRNA levels for the gene encoding the delta subunit of GABA_A_ receptor subunit—specific to the extrasynaptic population of receptors—has been reported to be reduced (Gantois et al., 2006). In terms of GABAergic function, release of synaptic GABA was reduced and phasic and tonic inhibition diminished in the amygdala (Olmos-Serrano et al., 2010).

With regard to behavior, hyperactivity, auditory startle, and learning and memory observed in FX mice have been correlated with the loss of inhibitory tone from GABAergic inputs into the cerebellum and amygdala (Olmos-Serrano et al., 2010), two brain regions also linked to human FXS (Lightbody and Reiss, 2009; Hall et al., 2013).

More critically, selective potentiation of delta-subunit-containing extrasynaptic GABA_A_ receptors (dSEGAs) with gaboxadol, also known as OV101 and 4,5,6,7-tetrahydroisoxazolo(5,4-c)pyridin-3-ol (THIP), normalized aberrant hyperactivity and auditory startle responses in the *Fmr1* KO mice (Olmos-Serrano et al., 2010). Importantly, these results were observed at exposures equivalent to those at which gaboxadol is well-tolerated in humans (Meera et al., 2011).

Because initial clinical trials have yet to show any therapeutic benefit of mGluR5 inhibitors (Scharf et al., 2015), in the present study, we sought to explore approaches that could more directly inform a clinical plan, including wider aspects of the FXS phenotype not previously reported. Specifically, here we investigated the effects of various doses of gaboxadol on hyperactivity, anxiety, aggression, and repetitive behaviors in the *Fmr1* KO2 model of FXS, demonstrating the efficacy potential of gaboxadol for treating those FXS symptoms.

## Materials and Methods

### Animals and Housing

*Fmr1* KO2 mice, generated previously by the deletion of the promoter and first exon of *Fmr1* resulting in mRNA and protein null mice (Mientjes et al., 2006), were used for all experiments. Floxed *Fmr1* mice were bred to mice expressing an early ubiquitously expressed Cre-recombinase resulting in the elimination of *Fmr1* from all cells. KO mice were backcrossed for at least eight generations to C57BL/6J, and wild-type-littermates (WT) were used as controls. Ten mice (males, 2 months of age) were used for each treatment group across all behavioral experiments. Heterozygous breeding pairs were used to generate WT and KO littermates for all studies. Males were removed from the breeder cages after birth, females were culled from the litters and genotyping was performed using TransnetXY Automated Genotyping (Transnetyx, Inc., Cordova, TN, USA) using established primers and protocol. Mice in the same cage were injected with the same dose of gaboxadol or vehicle, and mutants and controls were housed separately. All mice were group-housed in plastic cages (35 × 30 × 12 cm), five per cage, and habituated to the animal facility for at least a week before testing. The room temperature (21 ± 2°C), relative humidity (55 ± 5%), a 12 h light-dark cycle (lights on 7 am–7 pm) and air exchange (16 times per hour) were automatically controlled. All mice had *ad libitum* access to food and water. All testing was conducted in the light-phase by an investigator blind to genotype and drug treatment. Housing and experiments took place at GeN DDI Limited (London, UK) in accordance with the requirements of the UK Animals (Scientific Procedures) Act of 1986.

### Gaboxadol Treatment and Experimental Timeline

*Fmr1* KO2 mice were injected with vehicle (0.9% sterile saline) or gaboxadol (0.5, 1, 1.5, 2, 3, 4, or 5 mg/kg, i.p.) 30 min prior to behavioral testing on each testing day, with a three-day interval between each test to avoid any cumulative effect of the drug administration. Wild-type mice injected with vehicle at the same time point were also included in all experiments. Behavioral screening of the mice (*n* = 10 per group) was conducted in the following order with 2–3 days between each test: Open Field Test (OFT; day 1), successive alleys (day 4), light/dark box (day 7), social tests and aggression (day 10), and self-grooming and stereotypy (day 12).

### Open Field Test (OFT)

On testing day 1, the OFT was conducted in a VersaMax activity monitor chamber (AccuScan Instruments, Columbus, OH, USA). Locomotor activity was recorded for 30 min in a large Plexiglas chamber (40 × 40 cm) illuminated at 40-lux. Activity was monitored by infrared beam breaks that were combined into 1-min bins and decoded using VersaDat software (AccuScan Instruments, Columbus, OH, USA) to determine the distance traveled (cm), center distance traveled (cm), clockwise and counterclockwise revolutions (of at least 5.08 cm in diameter), and repetitive beam breaks.

### Successive Alleys Test (SAT)

On testing day 4, mice were subjected to the successive alleys test (SAT). The SAT has an advantage over the elevated plus maze as it brackets a wider range of anxiety levels (Deacon, 2013). The testing chamber consisted of four successive, linearly arranged, increasingly anxiogenic (lighter color, lower walls, narrower) alleys that were 25 cm long and 50 cm high-off the ground. Mice were observed over a 5-min period under a 60-W red light positioned above the apparatus. The number of entries into each alley was recorded.

### Light-Dark Exploration Test (LDT)

On testing day 7, mice underwent the light/dark exploration test (LDT). The LDT is widely used to measure anxiety-like behavior in mice based on their innate fear of bright, open spaces (Prut and Belzung, 2003). The polypropylene testing chamber (44 × 21 × 21 cm) was divided into a smaller, dark, covered compartment (14 cm) and a larger, brightly lit, open compartment (28 cm) by a dark partition with a small opening (13 × 5 cm). Transitions between the compartments were recorded by photocells located in the partition opening. Each mouse was placed in the light side facing away from the partition, allowed to freely explore the chamber for 10 min, and transitions between chambers were recorded.

### Social Interaction (SI), Aggressive Behaviors

On testing day 10, social interaction (SI) and aggression testing was performed in a cage similar in size to the home cage (40 × 23 × 12 cm) with a Perspex lid to facilitate viewing of the mice. Mice were habituated to the testing room for 25 min. Two subjects, an experimental mouse and a wild-type “test” mouse, were then placed in the testing cage simultaneously. The total duration and number of social investigations, tail rattling, number of bites, and number of mounts were recorded from above and measured for 3 min.

### Stereotypy

On testing day 12, rates of spontaneous stereotypy (head movements) were assessed using a modified automated photocell apparatus (Columbus Instruments, Columbus, OH, USA). The testing protocol involved removing the mice from their home cages and placing them singly in Plexiglas testing cages (22 × 25 × 28 cm). The mice were left undisturbed for 2 min for habituation to the testing chamber. Then, each mouse was monitored for 3 min to generate a stereotypy score that corresponds with the average stereotypy frequency per hour. Food and water were provided throughout the testing period. The stereotypy count is a quantification of the number of times a mouse interferes with the same infrared beam in a bout of stereotypic activity as measured by the testing apparatus.

### Self-grooming

Also on testing day 12, self-grooming was monitored. Each mouse was placed individually into a standard mouse cage (46 × 23.5 × 20 cm) illuminated at 40-lux. After a 5-min habituation period in the test cage, cumulative time spent grooming any body region was recorded for 3 min to determine the cumulative time (in seconds) grooming any body region.

### Statistical Analysis

Since *Fmr1* KO2 mice were treated with gaboxadol or vehicle, but WT mice were only treated with vehicle, parametric data were analyzed using one-way analysis of variance (ANOVA) and Tukey’s *post hoc* test. All data were analyzed using GraphPad Prism v7.0 (GraphPad Software, San Diego, CA, USA). Statistical significance was set at *P* < 0.05.

## Results

### Gaboxadol Normalizes Hyperactivity Observed in *Fmr1* KO2 Mice

**Figure 1:**
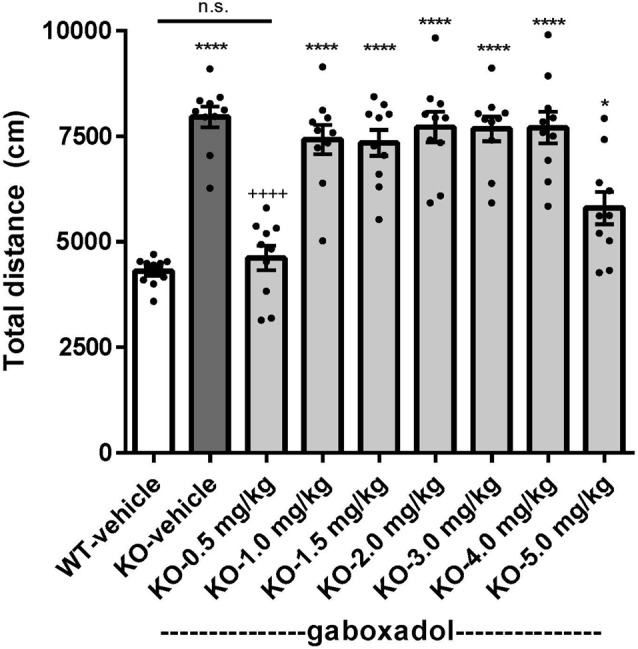
Gaboxadol normalizes hyperactivity in *Fmr1* KO2 mice. Total distance traveled for 30 min for wild-type-littermates (WT) mice treated with vehicle (white bar), *Fmr1* KO2 mice treated with vehicle (knockout, KO dark gray bar) and *Fmr1* KO2 mice treated with gaboxadol (KO-0.5–5.0 mg/kg, light gray bars). Bars are means ± SEM, dots are raw data from individual mice. *p < 0.05, ****p < 0.0001 vs. WT-vehicle group; ns, not significant vs. WT-vehicle group; ^++++^*p* < 0.0001 vs. *Fmr1* KO2-vehicle group. *N* = 10 per group.

Hyperactivity is a salient feature of human FXS (Bailey et al., 2008; Wheeler et al., 2014; Hagerman et al., 2017) and has been reliably reproduced in the previously characterized Dutch-Belgian *Fmr1* KO mouse (Olmos-Serrano et al., 2010; Kazdoba et al., 2014). To test whether the *Fmr1* KO2 mice showed locomotor hyperactivity and whether gaboxadol could normalize this aberrant behavior, *Fmr1* KO2 mice were injected with vehicle or gaboxadol (0.5–5 mg/kg, i.p.), and WT littermates were injected with vehicle 30 min before testing in the OFT. The total distance traveled (cm) in the OFT was recorded for 30 min. The results showed that the distance traveled by *Fmr1* KO2 mice was significantly increased compared to WT littermate controls (Figure 1, *F*_(8,81)_ = 21.27, *p* < 0.0001), consistent with results from other models of FXS. Treatment with gaboxadol (0.5 mg/kg) normalized the distance traveled by *Fmr1* KO2 mice to WT activity levels (Figure 1). Higher doses of gaboxadol (1–5 mg/kg, i.p.) had no effect on locomotor activity in *Fmr1* KO2 mice (Figure 1). These results were not attributable to sedative effects of gaboxadol because in WT C57Bl/6 or BALB/c mice, gaboxadol doses up to 2.0 mg/kg, i.p. have no effect on locomotor activity in a 60 min OFT (data not shown), consistent with previous work showing no effect of gaboxadol on locomotion in WT mice (Olmos-Serrano et al., 2011) or rats (Silverman et al., 2016).

### Anxiety-Like Behaviors in *Fmr1* KO2 Mice Are Normalized by Gaboxadol

**Figure 2:**
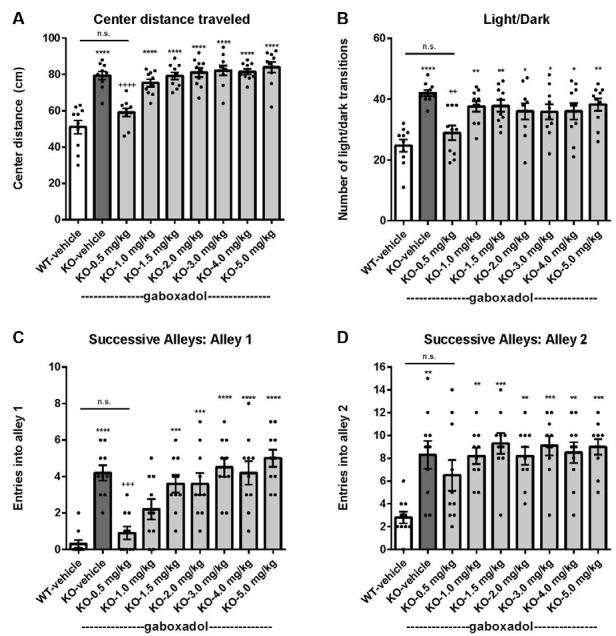
Gaboxadol normalizes anxiety-related behaviors in *Fmr1* KO2 mice. WT mice treated with vehicle (white bar), *Fmr1* KO2 mice treated with vehicle (KO, dark gray bar), or *Fmr1* KO2 mice treated with gaboxadol (KO-0.5–5.0 mg/kg, light gray bars) were subjected to the open field test (OFT), light/dark exploration test (LDT), and successive alleys test (SAT). **(A)** Total distance traveled in the center of the OFT. **(B)** The number of transitions between light and dark compartments in the LDT. **(C)** The number of entries into Alley 1 during the SAT. **(D)** The number of entries into Alley 2 during the SAT. Bars are means ± SEM, dots are raw data from individual mice. ****p < 0.0001, ***p < 0.001, **p < 0.01, *p < 0.05 vs. WT-vehicle group; ns, not significant vs. WT-vehicle group; ^++++^*p* < 0.0001, ^+++^*p* < 0.001, ^++^*p* < 0.01 vs. *Fmr1* KO2 vehicle group. *N* = 10 per group.

To assess the effect of gaboxadol on anxiety-like behaviors in the *Fmr1* KO2 mice, three different behavioral tests were employed: center distance traveled in the OFT, the LDT and the SAT. Increased distance traveled in the center is interpreted as decreased anxiety and takes advantage of the inherent preference of mice to remain in the perimeter when introduced to a novel environment. *Fmr1* KO2 mice were injected with gaboxadol (0.5–5 mg/kg, i.p.), and WT littermates were injected with vehicle 30 min before being placed in the OFT for 30 min. The total distance traveled in the center was significantly increased in *Fmr1* KO2 mice compared to WT controls (Figure 2A, *F*_(8,81)_ = 21.32, *p* < 0.0001). Treatment with gaboxadol (0.5 mg/kg, i.p.) normalized the effect of *Fmr1* KO2 on center distance traveled to levels comparable to WT controls (Figure 2A). Higher doses of gaboxadol (1–5 mg/kg) had no effect on *Fmr1* KO2 mice in this test (Figure 2A).

Next, the LDT, which takes advantage of the natural preference of mice for dark, protected environments, was used as another behavioral assay for anxiety. Willingness to explore the light compartment of the chamber, measured by the number of transitions between compartments, is interpreted as anxiolytic behavior and is sensitive to treatment with anxiolytic agents (Bourin and Hascoët, 2003). *Fmr1* KO2 mice showed a significantly increased number of transitions between chambers in the LDT compared to WT mice (Figure 2B, *F*_(8,81)_ = 5.819, *p* < 0.0001). Gaboxadol (0.5 mg/kg, i.p.) normalized the behavioral phenotype of the *Fmr1* KO2 mice to WT levels (Figure 2B). Higher doses of gaboxadol had no effect on the light/dark transitions of the *Fmr1* KO2 mice (Figure 2B).

Finally, results from the SAT reinforced that the anxiety-related phenotype observed in *Fmr1* KO2 mice could be normalized by gaboxadol treatment. This test is used as a more sensitive variant of the Elevated Plus Maze, and is comprised of four linear, successive, increasingly anxiogenic alleys (Deacon, 2013). The number of entries into alleys 2–4 assesses anxiety behavior (Deacon, 2013), whereas increased entries into alley 1 are consistent with general hyperactivity (Deacon, 2013). Importantly, previous work has shown that locomotor hyperactivity does not lead to false positive results in this test (reviewed in Deacon, 2013). *Fmr1* KO2 mice showed an increased number of entries into alley 1 (Figure 2C, *F*_(8,81)_ = 11.50, *p* < 0.0001), consistent with increased locomotor activity and the hyperactivity phenotype shown in Figure 1. *Fmr1* KO2 mice also showed a significantly increased number of entries into alley 2 compared with WT controls (Figure 2D, *F*_(8,81)_ = 4.925, *p* < 0.0001), supporting an anxiety-related phenotype. Injection of gaboxadol (0.5 mg/kg) into *Fmr1* KO2 mice 30 min before testing normalized entries into Alleys 1 and 2 to the levels of WT controls (Figures 2C,D). Higher doses of gaboxadol had no effect on the *Fmr1* KO2 mice in the SAT (Figures 2C,D). Entries into Alleys 3 and 4 were significantly increased in *Fmr1* KO2 mice relative to WT controls, but no obvious benefit of gaboxadol was seen with any dose tested (not shown).

### Irritability and Aggressive Behaviors in *Fmr1* KO2 Mice Are Normalized by Gaboxadol

As with other forms of syndromic autism, a large proportion of individuals with FXS show irritability, social anxiety and aggression. These aberrant behaviors can be modeled in rodents through characterization of SIs between a test mouse and a novel cage-mate. To test the hypothesis that irritability and aggression were increased in *Fmr1* KO2 mutants, we quantified instances of tail rattling, biting behavior, mounting behavior, and latency to attack. Mice were injected with vehicle or gaboxadol (0.5–5 mg/kg, i.p.) 30 min before being placed into the test cage.

**Figure 3:**
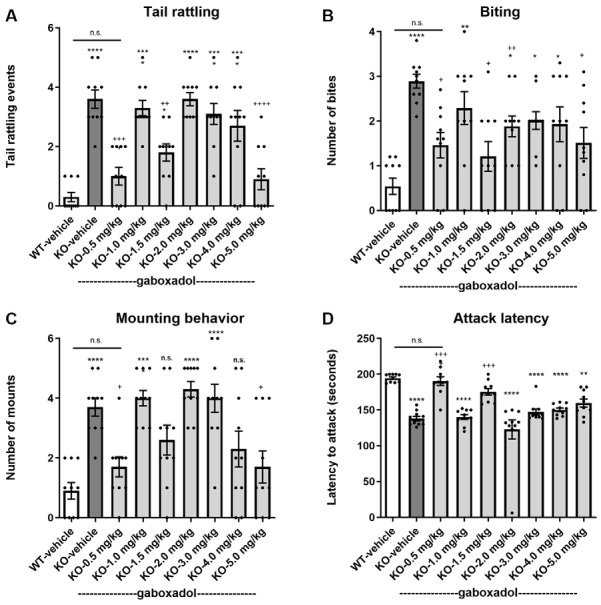
Gaboxadol normalizes irritability and aggression behaviors in *Fmr1* KO2 mice. Tail rattling **(A)**, biting behavior **(B)**, mounting behavior **(C)** and latency to attack a novel cage-mate **(D)** were measured in WT mice treated with vehicle (white bar), *Fmr1* KO2 mice treated with vehicle (KO, dark gray bar), or *Fmr1* KO2 mice treated with gaboxadol (KO-0.5–5.0 mg/kg, light gray bars). Bars are means ± SEM, dots are raw data from individual mice. ****p < 0.0001,***p < 0.001,**p < 0.01, *p < 0.05 vs. WT-vehicle group; ns, not significant vs. WT-vehicle group; ^++++^*p* < 0.0001, ^+++^*p* < 0.001, ^++^*p* < 0.01, ^+^*p* < 0.05 vs. *Fmr1* KO2 vehicle group. *N* = 10 per group.

Tail rattling, or rapid vibrations of the tail, reflects aggressiveness and fight tendency. *Fmr1* KO2 mice showed significantly increased tail rattling frequency compared to WT controls (Figure 3A, *F*_(8,81)_ = 16.03, *p* < 0.0001). Gaboxadol (0.5, 1.5, and 5.0 mg/kg) normalized the effect in *Fmr1* KO2 mice to levels comparable to WT controls (Figure 3A).

Like tail rattling, biting is a measure of aggression in mice. *Fmr1* KO2 mice showed a significantly increased number of bites compared to WT controls (Figure 3B, *F*_(8,81)_ = 5.446, *p* < 0.0001). Gaboxadol (0.5, 1.5, and 5.0 mg/kg) significantly decreased the number of bites made by *Fmr1* KO2 mice (Figure 3B). However, at some of the doses tested (1.0, 3.0, and 4.0 mg/kg), biting behavior remained significantly increased compared to WT-vehicle groups and was not significantly changed compared to the KO-vehicle controls (Figure 3B).

In male mice, mounting behavior is an aggressive assertion of social dominance. *Fmr1* KO2 mice showed a significantly increased number of mounts compared to WT controls (Figure 3C, *F*_(8,81)_ = 9.008, *p* < 0.001). Treatment with gaboxadol (0.5, 1.5, 4.0, and 5.0 mg/kg) significantly decreased mounting behavior in the *Fmr1* KO2 mice, and the effects of 0.5 and 5.0 mg/kg doses were statistically significant compared to KO-vehicle treated controls (Figure 3C). In *Fmr1* KO2 mice treated with 1.5 and 4.0 mg/kg gaboxadol, mounting behavior did not differ significantly from WT-vehicle treated mice, suggesting a trend for an effect at these doses even though their comparison to the KO-vehicle treated group did not reach statistical significance (Figure 3C).

Finally, the latency to attack a newly encountered mouse was recorded as another measure of aggression. *Fmr1* KO2 mice showed a significantly decreased latency to attack compared to WT littermates (Figure 3D, *F*_(8,81)_ = 17.22, *p* < 0.0001). The reduced latency to attack was normalized in *Fmr1* KO2 mice treated with gaboxadol (0.5, 1.5 mg/kg; Figure 3D).

### Gaboxadol Normalizes Repetitive Behaviors in *Fmr1* KO2 Mice

**Figure 4:**
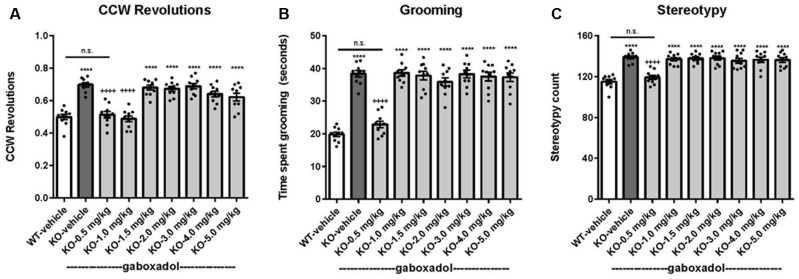
Gaboxadol normalizes repetitive behaviors in *Fmr1* KO2 mice. WT mice treated with vehicle (white bar), *Fmr1* KO2 mice treated with vehicle (KO, dark gray bar), or *Fmr1* KO2 mice treated with gaboxadol (KO-0.5–5.0 mg/kg, light gray bars) were subjected to four tests that measure repetitive behaviors. **(A)** Counter-clockwise (CCW) revolutions were measured by infrared beam breaks during a 5-min test in the Open Field. **(B)** After a 5-min habituation to the test cage, time spent grooming for 3 min is shown. **(C)** Stereotypy counts (head bobbing events) during a 3-min testing period are shown. Bars are means ± SEM, dots are raw data from individual mice. ****p < 0.0001 vs. WT-vehicle group; ns, not significant vs. WT-vehicle group; ^++++^*p* < 0.0001 vs. *Fmr1* KO2 vehicle group. *N* = 10 per group.

Perseveration and repetitive behaviors are common in individuals with FXS and are highly disruptive (Arron et al., 2011; Leekam et al., 2011; Hall et al., 2016). To test the hypothesis that such features might be observed in *Fmr1* KO2 animals, we quantified circling, self-grooming, and stereotypy in WT and *Fmr1* KO2 mutant mice. Counter-clockwise (CCW) revolutions were measured in the testing chamber after mice were injected with vehicle or gaboxadol (0.5–5 mg/kg, i.p.). *Fmr1* KO2 mice showed significantly increased CCW revolutions during the 5 min test compared with WT controls (Figure 4A, *F*_(8,81)_ = 25.46, *p* < 0.0001). Injection of gaboxadol (0.5, 1.0 mg/kg) into *Fmr1* KO2 mice restored the number of CCW revolutions to WT levels (Figure 4A). There was no effect of genotype on clockwise circling (*p* = 0.386, data not shown).

The time spent grooming was significantly increased in the *Fmr1* KO2 mice compared to WT controls (Figure 4B, *F*_(8,81)_ = 41.99, *p* < 0.0001). In *Fmr1* KO2 mice, gaboxadol injection (0.5 mg/kg) normalized time spent grooming to WT levels (Figure 4B).

Stereotypy is defined as repetitive and ritualistic movements and is commonplace in autism and FXS. In mice, stereotypic activities such as head bobbing can be measured by quantifying infrared beam breaks in the test cage. Stereotypy counts were significantly increased in *Fmr1* KO2 mice compared to WT controls (Figure 4C, *F*_(8,81)_ = 19.93, *p* < 0.0001). *Fmr1* KO2 mice injected with gaboxadol (0.5 mg/kg) had normalized stereotypy behavior back to WT levels (Figure 4C).

## Discussion

**Figure 5:**
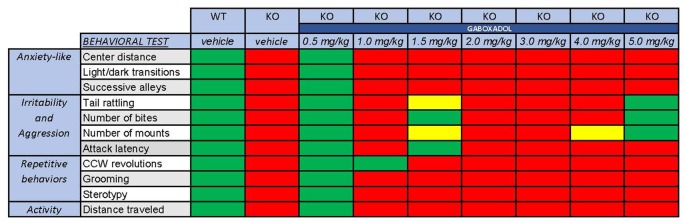
Overall behavioral effects of gaboxadol in *Fmr1* KO2 mice. Summary of the results of behavioral assessment of *Fmr1* KO2 mice treated with vehicle or gaboxadol (0.5–5.0 mg/kg) compared to WT littermate controls. The *Fmr1* KO2 mice showed significant phenotypes in locomotor activity, anxiety-related behaviors, irritability and aggression, and repetitive behaviors. All of these phenotypes were consistently normalized to WT levels by treatment with gaboxadol at the 0.5 mg/kg dose. “Normal” behavior (green squares) is consistent with WT vehicle-treated mice and is statistically different from *Fmr1* KO2 (KO) vehicle-treated mice. “Abnormal” behavior (red squares) is consistent with *Fmr1* KO2 (KO) vehicle-treated mice and is statistically different from WT vehicle-treated mice. Yellow squares indicate a difference that is *either* not significantly different from WT mice *or* statistically different from *Fmr1* KO2 (KO) vehicle-treated mice.

Clinical symptoms of FXS can include hyperactivity, anxiety, memory and learning deficiencies, social abnormalities, aggression and repetitive behaviors. In all cases, treatment with 0.5 mg/kg gaboxadol restored the behavior of the *Fmr1* KO2 mice to WT levels (summarized in Figure 5). These data provide additional support for augmentation of dSEGA activity and tonic inhibition by gaboxadol as a therapeutic strategy that warrants further investigation in individuals with FXS.

Locomotor hyperactivity is a feature of human FXS (Bailey et al., 2008; Wheeler et al., 2014; Hagerman et al., 2017). In the current study, *Fmr1* KO2 mice showed increased locomotor activity that was normalized by gaboxadol. Previous work in the Dutch-Belgian *Fmr1* KO mice has also shown a consistent hyperactivity phenotype that was reversed with gaboxadol treatment, however at a higher dose (Olmos-Serrano et al., 2011). Potential explanations for this difference include: differential regulation of *Gabrd* mRNA (D’Hulst et al., 2006; Curia et al., 2009), altered surface expression of extrasynaptic GABA receptors (Zhang et al., 2017), effects on brain structure (Lai et al., 2016), or differences in the behavioral manifestations of the mutation (Pietropaolo et al., 2011; Spencer et al., 2011). Each could be mediated by the result of genetic background, as previous work was done using FVB mice whereas animals studied here were C57BL/6J. Subtle differences in laboratory equipment, animal handling, and even housing conditions, which are well-established modifiers of mouse behavior, may also contribute (Crabbe et al., 1999; Wahlsten et al., 2006).

The results from three anxiety tests, center distance in the OFT, light/dark box, and SAT, demonstrate a robust phenotype in the *Fmr1* KO2 mouse that is reversed by treatment with gaboxadol. It should be noted that hyperactivity could confound the OFT and light/dark box results. However, anxiety-related behavior observed in the SAT occurs independent of locomotor activity changes (reviewed in Deacon, 2013); and while the anxiety effect in the KO is in the opposite direction of what is observed in patients with FXS (Bailey et al., 2008), our results are consistent with previous reports describing the FXS mouse phenotype (reviewed by Kazdoba et al., 2014). Nevertheless, it will be important that future studies aim to further elucidate the anxiety effect in these mice. Despite this, and most notably, our data demonstrate that gaboxadol normalizes the aberrant anxiety behavior observed in the FXS mouse model as well as in all the other behaviors assessed in our analyses.

Irritability and aggressive behaviors, modeled by measuring SIs with a novel cage mate, were also increased in the *Fmr1* KO2 mice and reversed by gaboxadol. A recent study reported that nearly all (>90%) males and females with FXS surveyed engaged in some aggression in the previous 12 months. Among them, 33% of males and 20% of females showed severe aggression, enough to cause injury to care givers (Wheeler et al., 2016). Self-injury and impulsive behavior are also more prevalent in individuals with FXS (Arron et al., 2011).

Repetitive behaviors and stereotypy were consistently increased in *Fmr1* KO2 mice and alleviated by gaboxadol treatment. Repetitive behaviors can cause serious problems for daily functioning and can become a barrier to learning and SI (Leekam et al., 2011). Paradigms that assess stereotypic behaviors and socioemotional deficits might thus be useful to assess novel drugs for their effectiveness to ameliorate the autistic phenotypes in FXS.

Gaboxadol normalized all of the tested behavioral deficits of *Fmr1* KO2 mice at a dose of 0.5 mg/kg. While higher doses also normalized irritability and aggressive behaviors, this was not observed for other behavioral domains evaluated. One explanation for the somewhat narrow efficacy window observed here may come from previous work showing compromised information processing by either insufficient or excess tonic inhibition, the physiological process that gaboxadol potentiates. Under this model, the behavioral benefit of drug at high doses would be offset by pharmacologically introduced FXS-independent deficits (Duguid et al., 2012).

Our results provide robust evidence of the potential benefit of gaboxadol in reversing ASD related behaviors, aggression and sociability. Taken together, these results support the hypothesis that potentiation of extrasynaptic GABA_A_ receptors by gaboxadol may be of benefit in individuals with FXS. In conclusion, these data support the future evaluation of gaboxadol in individuals with FXS, particularly with regard to symptoms of hyperactivity, anxiety, ASD related stereotypy, sociability, irritability, aggression, and cognition.

## Ethics Statement

All experiments were carried out under the approval of the Institute of Ecology and Biodiversity Ethical Committee, University of Chile, Santiago, Chile.

## Author Contributions

BA and MD initiated this work and worked with PC and RD to develop an experimental plan. PC and RD wrote a technical report summarizing results, which JW-S used to produce a manuscript ready for publication. MvS, like other authors, contributed to the interpretation of results. All authors provided feedback on the manuscript prior to submission.

## Conflict of Interest Statement

PC and RD are founder/CEO and CSO of Gen. DDI Limited, which was contracted by Ovid Therapeutics to carry out the experimental work described here. PC and RD are currently head of and team member of FRAXA-DVI. JW-S, through NeuroJenic Consulting, LLC, was contracted to draft this manuscript for publication. MvS, BA, and MD are full time employees at Ovid Therapeutics and also hold either stock or stock options.
